# Mental health nursing consultations in Brazilian primary care: analysis of proposed competencies for advanced practice nurses

**DOI:** 10.1186/s12875-025-02761-w

**Published:** 2025-03-06

**Authors:** Patricia Aline de Almeida, Letícia Yamawaka de Almeida, Andrea Liliana Vesga-Varela, Carla Pereira Barreto, Marília Orlandelli Carrer, Keila Gisele Lima Reis, Nayara Vilela de Farias Serranegra, Manoel Vieira de Miranda Neto, Claudia Santos Martiniano, Daiana Bonfim

**Affiliations:** 1 Faculdade Israelita de Ciências da Saúde Albert Einstein (FICSAE), São Paulo, Brazil; 2 Hospital Israelita Albert Einstein, São Paulo, Brazil; 3 Universidade Estadual da Paraíba, Paraíba, Brazil

**Keywords:** Advanced nursing practice, Primary health care, Mental health, Professional competence, Nurse

## Abstract

**Background:**

Primary Health Care (PHC) is a key strategy to identify, manage, and coordinate mental health cases. Considering that nurses are essential to integrating mental health care into PHC, initiatives to broaden the discussion and incorporate the role of Advanced Practice Nurses in this setting can help reduce disparities in mental health care. Thus, this study aimed to analyze mental health nursing consultations in PHC and investigate whether nurses have the care management skills proposed for Advanced Practice Nurses.

**Methods:**

A multicenter study, with a quantitative and qualitative approach, was conducted in 17 Primary Care Health Units distributed in three regions of Brazil from May to July 2022. Data collection was carried out twice during the nurse’s professional practice: nursing consultation (recorded using film, with direct and non-participatory observation) and nursing record. From a quantitative perspective and during the first research stage, consultations that had ≥ 50% compliance with the nursing process were selected so that, in the second qualitative stage, the competencies proposed for Advanced Practice Nurses in PHC were identified through content analysis.

**Results:**

A total of 49 mental health nursing consultations were performed by 21 nurses. Of these, seven were selected with a score greater than 50% compliance with the nursing process, carried out by three nurses. The consultations presented few competencies in the care management dimension proposed for the Advanced Practice Nurses; nevertheless, nursing consultation presented 39.68% in the care focus, 38.78% in evaluation and diagnosis, and 47.62% in the provision of care.

**Conclusions:**

Nurses who conduct mental health nursing consultations in PHC present, in a scarce and partial way, the competencies proposed for the Advanced Practice Nurses for the care management domain. Hence, the results of this study highlight the need for specific training and policy initiatives to enhance the integration of Advanced Practice Nurses in mental health care within PHC, address existing gaps in care management competencies, and improve the quality of mental health services provided to the population.

## Introduction

Mental disorders are among the leading causes of global disease burden [1]. Despite this, significant gaps remain between the demand for and the availability of mental health care worldwide. According to the World Health Organization, the treatment gap exceeds 70% in many countries [2].

In Brazil, advancements in mental health care in Brazil are largely the result of processes initiated by the psychiatric reform [3] that recently culminated in the creation of the Psychosocial Care Network (RAPS) [4], which replaced the asylum model and provides care for individuals with mental disorders and those with needs related to alcohol and other drug use through an integrated and coordinated set of community-based and territorial services.

Grounded in a commitment to guaranteeing human rights, these services encourage the social participation of users and their families while adopting a comprehensive approach to health and illness [4]. From this perspective, the provision of care is not limited to mental health professionals. Primary Health Care (PHC) teams often play a strategic role in identifying, managing, and monitoring mental health cases within the community.

Considering the significant prevalence of mental disorders in the population and the growing burden on healthcare systems [1], PHC teams must have organized processes and workflows to care for this portion of the population and operate based on the principles of health responsibility and territorialization. To achieve this, these teams must implement mental health care actions [5], employing strategies that consider multiple dimensions of the individuals experiencing mental illness, while respecting their values, choices, and desires [6].

In this context, the nurse can play a strategic role in integrating mental health care within PHC [7], with the nursing consultation being a promising component in this process, involving actions based on therapeutic interpersonal relationships and psychosocial intervention [8, 9]. Although some challenges have been reported in consolidating this care, promoting the performance of advanced roles [8, 11] by strengthening autonomy and expanding scope could be a strategy to increase access and address the needs of the population [12], particularly in resource-limited areas.

The focus has increased on Advanced Nursing Practices in several countries [13, 14]. Generally, this professional is defined as a nurse who presents complex decision-making skills and clinical competencies for expanded practice [15]. In practical terms, Advanced Nursing Practices mean that the nurse, in clinical settings, has a broader knowledge base and skill set. The nurse is legally empowered to assess, diagnose, prescribe, refer, and implement care plans. The key difference lies in greater autonomy in decision-making and problem-solving, which enhances the population’s access to qualified professionals and health services [13, 15].

The literature highlights experiences in advanced mental health practices in countries such as the USA [16], the United Kingdom [17], Austria [18], Spain [19], and Canada [20]. In Brazil, this discussion is relatively recent, and studies on Advanced Nursing Practices remain reflective and instrumental [21], with a particular gap related to mental health efforts, highlighting the need to investigate aspects of the practice that support the debate on implementing this professional role and presenting it as a resource that can collaborate with other strategies already in place to address gaps in mental health care provision.

Thus, considering PHC as an appropriate setting for psychosocial care, and recognizing Advanced Nursing Practices — an emerging field in Brazil, both from a regulatory perspective and in terms of knowledge advancement — as a potential strategy for expanding mental health care, the present study analyzed nursing consultations in mental health within PHC and investigated whether nurses possess the care management skills proposed for the Advanced Nursing Practices.

## Method

### Study design

**Fig. 1:**
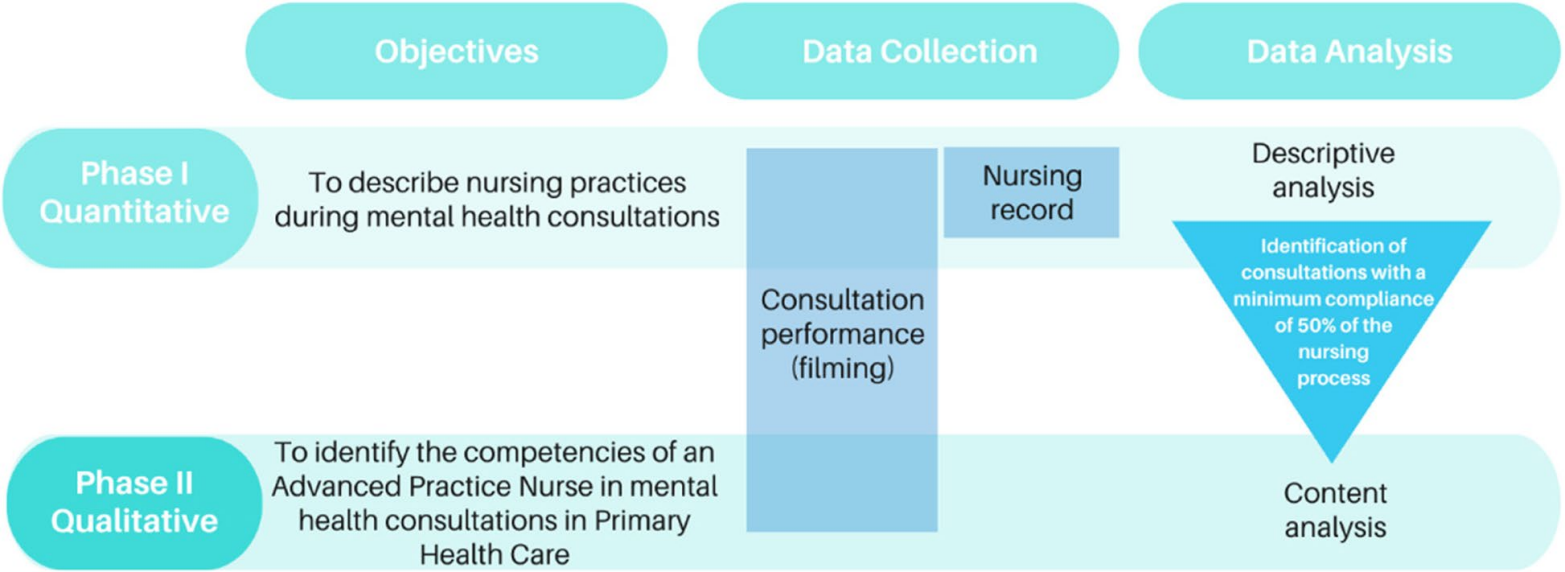
Representative diagram of the study design

This study employed both quantitative and qualitative approaches (Fig. 1). It is part of the major project entitled “Advanced practice skills in care management in primary care nursing consultations: situational diagnosis and training proposal,” which evaluates different lines of care for nurses in PHC. Research regarding mental health care will be presented here. The reporting of this study was guided by Consolidated Criteria for Reporting Qualitative Research [22] (COREQ) and Strengthening the Reporting of Observational studies in epidemiology (STROBE) [23] guidelines.

### Setting and data collection

Data collection was conducted in PHC services of four municipalities located in three Brazilian regions: six PHC units in the North (state of Amazonas – municipality A), seven PHC units in the Northeast (two services in Alagoas – municipality B – and five in Rio Grande do Norte – municipality C), and four PHC units in the Southeast (São Paulo – municipality D), totaling 54 Family Health Strategy teams located in 17 PHC units.

The selection of the municipalities for the sample was based on a convenience sampling approach. The choice was made due to the diversity in demographic profiles, morbidity, mortality, and service availability, which can provide the different dynamics of PHC in Brazil. These PHC services offer a workflow that combines scheduled appointments, such as the management of chronic conditions, with spontaneous care for acute conditions, ensuring comprehensive and equitable care for the population in the Brazilian Unified Public Health System.

To select the PHC units, a convenience sample was used, which occurred after the presentation of the research project to the municipalities, followed by an invitation to the nurses who worked in PHC units. The selection considered the number of nurses who agreed to participate in the research, as well as the number of family health teams, population covered, service structure, and demographic characteristics, which could promote practices and challenges of PHC in distinct contexts within Brazil. This initial stage of invitation and mapping of the PHC units that agreed to participate in the research was crucial as it allowed the establishment of an appropriate logistical plan, aligning the needs of the research with the operational realities of the health units. It also ensured that the selected units were prepared and able to participate in the study during data collection.

### Period

Data collection took place between May and July 2022.

### Study population and selection criteria

Nurses with at least one year of professional experience working in PHC units were included. They did not need to have a specialization in mental health to be included, as the objective of the research was to understand the dynamics of mental health care and consultations in different PHC settings, without restricting the participation of professionals with specific training. Nurse managers who did not perform direct care activities were excluded.

Healthcare users included were over 18 years of age who presented for a consultation with a nurse and had a demand for mental health care, characterized by a questionnaire administered by the researcher at the time of the invitation to participate in the research. A health professional must have confirmed their diagnosis for at least one of the following conditions: anxiety disorder, depressive disorder, and abuse of alcohol and/or other psychoactive substances. Additionally, consultations with users were included who reported, through the questionnaire, presenting at least three of the following nonspecific complaints, reflecting changes in the individual’s emotional and psychological well-being: multiple pains, malaise, nervousness, difficulty sleeping, palpitations, crying/sadness, discouragement, weakness, dizziness, tinnitus, chest tightness, and shortness of breath. Patients were excluded who presented an episode of a mental health crisis at the time of the consultation, characterized by a significant increase in motor activity and agitation involving restlessness, excessive movements, difficulty in remaining still, rapid speech, and impulsive and disorganized actions, which would not allow the application of free consent for participation in the research.

### Data collection

The methodological path for data collection was conducted at three different times: Pilot study; during the nursing consultation (collection through filming); and when the recorded consultation was registered in the medical record. The group of researchers who conducted this stage consisted of five previously trained nurses. The research team that carried out the data collection has diverse nursing educational backgrounds and experiences, reflecting a high level of specialization and academic qualification in the fields of PHC, mental health, and public health. These researchers were distributed across the four municipalities that participated in the data collection.

#### Pilot Study

The first phase of the pilot study was carried out in December 2021 at a Basic Health Unit in the city of São Paulo to verify the applicability of all the instruments developed for data collection and test the proposed data collection flow considering the patient approach, the positioning of the audiovisual material in the consultation room, and the completion of the instruments by the researchers. Thus, a pilot study was conducted with a nurse at a PHC unit in São Paulo. After the pilot, the applied questionnaires were reviewed, new questions were added, and another pilot was scheduled to address aspects related to the positioning of the camera in the consultation room and the capture of audio and video. Thus, in April 2022, a second pilot was done at a PHC unit in São Paulo. Analysis of the data collection dynamics and the necessary materials lead to the elaboration of a protocol to guide the researchers responsible for data collection, ensuring uniformity of the process. This protocol was called the “field data collection operationalization guide.”

#### Data collection at the time of nursing consultation: direct, non-participatory observation of nursing consultations through filming

Field preparation was carried out before data collection to capture nursing consultations through filming. For this, two researchers presented the research and detailed how the collection would occur for the nurses invited to participate. This took place in a group, online or in person, when appropriate. After clarifying doubts, the Informed Consent Form was presented to the nurses. Then, the researchers scheduled the time for data collection (initial data). The following instruments were applied to all nurses involved in this stage via the Research Electronic Data Capture (REDCap) platform: Informed Consent Form; Image and Voice Use Authorization Form; and characterization instrument.

During the recording of nursing consultations, two researchers (researchers A and B) were present to monitor data collection. Researcher A was responsible for inviting users in the waiting room who were waiting to see the nurse. After accepting and signing the terms, researcher A identified the users with colored bracelets (blue and orange), so that researcher B could consider the user as having accepted the Informed Consent Form before entering the office and thus activate the recording of the consultation. Researcher B was responsible for positioning the two recording cameras. One camera was placed in the office, to capture sound and image of the consultation, and the other, on the nurse’s body, to capture the interaction and content of the discussion of the case during the interconsultation with other professionals, when there was one. Thus, researcher B was responsible for positioning and activating the cameras, followed by the presentation of the user’s identification code (initials of their name, the numerical order of the consultation, and the initials of the municipality) and positioning of the lapel to capture the sound. When a consultation started, researcher B left the office. Then, when the user left the room, interrupting the end of the consultation, researcher B turned off the recording. At the end of a typical collection day, all records were securely stored in an institutional cloud connected to the Vimeo® platform. All recordings were made using GoPro® Hero9 cameras, 1080 k × 24 configuration, superview, 1x.

#### Data collection at the time of consultation records in medical records

To collect the medical records of the filmed consultations, after the end of the filming day, the researchers recorded the content of the consultation in a checklist. The data collection forms were developed and applied through the Research Electronic Data Capture (REDCap) data management platform.

### Data analysis

#### Quantitative analysis

To analyze mental health nursing consultations in PHC, a checklist was created in RedCap for data extraction, containing essential elements for conducting nursing consultations focused on mental health. This checklist was based on the five stages of the nursing process [24] and the Recommendations from the Mental Health Gap Action Programme Intervention Guide (mhGAP-IG) for mental, neurological, and substance use disorders in non-specialized health settings [25]. The checklist also included the “Calgary-Cambridge Guide to the Medical Interview – Communication Process” [26] to evaluate communication skills. Furthermore, a checklist (RedCap) for the extraction of medical records was developed, covering the stages of the nursing process [24].

An evaluation of the data was conducted, considering completeness and the verification of illogical values. Consultations were selected based on the inclusion and exclusion criteria established for mental health consultations.

To analyze the data, a univariate analysis was developed for all variables collected, using measures of central tendency, dispersion, and frequencies. The distribution of variables was assessed using the Kolmogorov–Smirnov test. Chi-square or Fisher’s test was performed to compare differences between groups, when necessary, and a *p*-value < 0.05 was adopted to identify statistically significant associations. Bivariate analysis was performed graphically to generate possible hypotheses regarding adherence to the nursing process.

The calculation of adherence to the nursing process was adjusted by the number of items evaluated in each stage. Thus, each stage was given equal weight in the percentage of adherence to the nursing process. Consultations with ≥ 50% adherence to the nursing process were selected for an in-depth analysis of the competencies proposed for Advanced Nursing Practices in PHC [27].

All statistical analyses were performed using R statistical software version 4.1.3 (R Project for Statistical Computing), together with RStudio statistical software version 1.4.1717 (RStudio).

#### Qualitative analysis

Content analysis was conducted to investigate whether nurses demonstrate the care management skills proposed for Advanced Nursing Practices [28]. The interpretation of results considered previously identified categories that refer to the dimensions comprising the skill profile proposed for Advanced Nursing Practices in PHC within the scope of care management [27].

This analysis used a guiding script consisting of three themes: focus on care; evaluation and diagnosis; and provision of care, along with 20 sub-items [27]. The material was explored systematically so that whenever a skill was highlighted, even partially, the scene and the dialogue between the nurse and the user were transcribed. At the end, the frequency of occurrence of each category was presented.

For the analysis of the collected data, the recordings of nursing consultations, which captured both image and sound, were transcribed by the lead author. The transcription process included not only the sound transcription of the audio but also a detailed description of the scenes to enable a broader and more contextualized analysis of the data. All material was meticulously reviewed to ensure fidelity to the original content, guaranteeing that no relevant details were omitted and preserving the integrity of the information obtained during the consultations.

In addition to the recorded conversation, non-verbal interactions, such as facial expressions, gestures, and other visual elements, were described, enriching the qualitative analysis. The transcription process was conducted by researchers reflexively, undergoing reviews and reflections to ensure that interpretations and categorizations respected the contextual and cultural nuances observed in the scenes.

To ensure confidentiality and adhere to ethical principles, the identities of all participants were protected through anonymization, using alphanumeric codes to identify consultations in the transcribed text.

These procedures were implemented to ensure the quality and credibility of the study, guaranteeing that the results accurately reflect the interactions observed in the nursing consultations.

### Ethical aspects

The study was approved by the Research Ethics Committee of Hospital Israelita Albert Einstein (CAAE: 56,255,622.2.0000.0071) and the co-participating Centers. All data is password protected on the institutional cloud software connected to the Vimeo® platform and RedCAP, and only researchers registered with the Ethics Committee approved had access to the data during the study.

## Results

A total of 203 nursing consultations were recorded. However, based on the eligibility criteria, only 49 consultations were included in the present study, distributed in municipality A (30.6%), municipality B (12.2%), municipality C (2.0%), and municipality D (55.1%), with the predominance of these consultations being evidenced in one of the regions investigated. These consultations were conducted by 21 nurses and involved 49 users.

The healthcare users were an average age of 41 years (± 15.9), 85.7% (*n* = 42) were female, 64.5% (*n* = 31) identified as mixed race, and 44.9% (*n* = 22) had completed high school. Furthermore, most of them were residents of urban areas (91.6%; *n* = 44), were not working or were unemployed (53.0%; *n* = 26), declared that they did not have partners (61.2; *n* = 30), and had children (83.7%; *n* = 41). When asked about their mental health, 26.5% of service users reported having a diagnosis of anxiety and/or depression and 4.1% reported abusing drugs and/or alcohol. Moreover, 95.9% referred to three or more nonspecific symptoms, with the most common being difficulty sleeping (63.2%).

The personal characteristics questionnaire was completed by 20 of the 21 nurses who participated in the study. Of these, the majority were female (75.0%), self-identified as mixed race (60.0%), and earned more than seven minimum wages (60.0%), some holding an additional job outside of PHC settings (35.0%). At the time, the studied nurses were working in units with the Family Health Team (65.0%) and the multidisciplinary team (80.0%). Nine nurses reported having more than ten years of experience in PHC (45.0%).

The academic background of all these nurses (100%) included a postgraduate specialization in PHC (35.0%), Family and Community Health, Public Health (45.0%), Mental Health or Psychiatry (10.0%), or another area (70.0%). Moreover, some nurses had completed a professional master’s degree (5.0%) or academic master’s degree (5.0%). Many of the nurses reported taking a training program in the last year; specifically, 10.0% of them reported having studied Mental Health and 30.0% the nursing process.

When asked about conducting a nursing consultation, the majority of the nurses reported experiencing some difficulty in carrying out the consultation (70.0%). They mentioned their lack of standardized instruments for performing the consultation (60.0%) despite reporting the existence of nursing protocols in their context (60.0%).

The median time spent in the analyzed consultations was 20 min, with a high percentage of interruptions (≅ 59.2%) due to demands related to the search for a nurse or other reasons. The satisfaction reported by users with the service was, on average, 9 out of 10.

Nursing practice in mental health consultations at PHC, based on the steps of the nursing process, is described in Table 1. In general, gaps were observed in the execution of the nursing process steps and accurate recording.

**Table 1:** Description of compliance with the nursing process steps in a mental health consultation in PHC. Brazil, 2023

Nursing process steps	Absent	Present
	**n(%)**	**n(%)**
**History**		
Is the first reason mentioned by the user related to Mental Health?	44 (89.8)	5 (10.2)
At any point, did the nurse inquire about or address Mental Health complaints?	38 (77.5)	11 (22.4)
At any point, did the user mention a mental health complaint/diagnosis?	35 (71.4)	14 (28.5)
Does the nurse employ clinical reasoning to identify the factors related to the Mental Health complaint?	44 (89.8)	05 (10.2)
Did the nurse inquire about daily stressors?	42 (85.7)	07 (14.2)
If yes, did the nurse investigate the psychosocial context?	44 (89.8)	05 (10.2)
Did the nurse perform a physical examination? (This may be partial)	12 (24.4)	37 (75.5)
Did the nurse conduct a psychological examination?	49 (100)	00
Did the nurse inquire about the user’s history of Mental Health conditions?	46 (93.8)	03 (6.1)
Did the nurse address continuity of care for Mental Health conditions?	43 (87.7)	06 (12.2)
Did the nurse inquire about the use of psychotropic medications?	44 (89.8)	5 (10.2)
**Nursing Diagnosis**		
Did the nurse express any nursing diagnosis?	47 (95.9)	02 (4.0)
**Planning**		
Did the nurse agree on the care plan with the user?	08 (16.3)	41 (83.6)
Did the nurse make a nursing prescription?	26 (53.0)	23 (46.9)
**Implementation**		
Did the nurse apply elements of psychosocial intervention?	44 (89.8)	05 (10.2)
Does the user require a referral or assistance from another professional? (observer evaluation)	46 (93.8)	03 (6.1)
Did the nurse refer the user to a physician?	47 (95.9)	02 (4.0)
Did the nurse refer the user to a Multiprofessional Team?	48 (97.9)	01 (2.0)
Did the nurse request matrix support from the Multiprofessional Team?	49 (100)	00
Did the nurse refer the user to other services?	47 (95.9)	02 (4.0)
Did the nurse refer to Integrative and Complementary Health Practices?	40 (81.6)	09 (18.3)
Did the nurse carry out an interconsultation or request matrix support?	44 (89.8)	05 (10.2)
Does the nurse demonstrate comfort handling the Mental Health demand?	44 (89.8)	05 (10.2)
Did the nurse address aspects of general health promotion?	27 (55.1)	22 (44.9)
**Evaluation**		
Does the nurse mention the need for a follow-up meeting?	28 (57.14)	21 (42.86)

After analyzing the nursing process steps, aspects related to clinical communication were evaluated. The nurse greeted and/or identified the user in 83.6% (*n* = 41) of consultations; introduced themselves in 18.3% (*n* = 9); paid attention to the user’s comfort or privacy in 59.1% (*n* = 29); started the consultation with open questions in 48.9% (*n* = 24); alternated the use of open and closed questions throughout the consultation in 40.8% (*n* = 20); maintained eye contact during interaction with the user in 65.3% (*n* = 32); and allowed enough time for the user to express themselves without interrupting them in 67.3% (*n* = 33).

Furthermore, they adapted their wording, avoiding the use of scientific language in 85.7% (*n* = 42); avoided criticism or value judgments of the user in 86.6% (*n* = 41); avoided non-verbal communication that denotes disinterest or disapproval in 65.3% (*n* = 32); repeated the user’s message validating their understanding and information in 38.7% (*n* = 19); and verified the user’s understanding by encouraging the clarification of doubts in 40.8% (*n* = 20). They ended the consultation formally indicated by a parting look or expression in 61.2% (*n* = 30) and summarized the information and prioritized the patient’s needs in 32.7% (*n* = 19).

However, active listening was still rarely present in the consultations, as nurses only paid attention 26.5% (*n* = 13) of the time to the user’s verbal and non-verbal manifestations. Other aspects rarely observed were the user’s involvement in planning actions and discussing therapeutic options (16.3%; *n* = 8) and in the summarization of the main points addressed during the consultation (10.2%; *n* = 5). Encouraging the user to verbalize feelings, expectations, and/or concerns was verified in only 18.3% (*n* = 9) of the consultations, and feedback was never requested on the user’s understanding of what was discussed in the meeting.

Nursing records were found for 87.7% (*n* = 43) of the consultations. The existing records were not sufficiently complete, with the presence of subjective data in 51.1% (*n* = 22) and objective data in 76.7% (*n* = 33). As observed in the videos, only 44.1% (*n* = 19) of the nursing records included a description of the physical examination, and the psychological examination was not recorded by any of the nurses.

The nursing diagnoses step was absent in 51.1% (*n* = 22) of the nursing records analyzed. When present, only 20.9% (*n* = 9) were related to the health data collected through the nursing history, and 18.6% (*n* = 8) partially responded. All nurses (100%) used the International Classification of Primary Care tool to record the reason for the patient’s consultation in the medical records. Of these, 86% (*n* = 37) of the records showed a consistent relationship between the reason for the consultation and the nursing history.

The analysis of the planning step of the nursing process focused on the documentation of the goals and expected outcomes, which were presented in only 6.9% (*n* = 3) of the nursing records. When considering the relationship between the described goals and outcomes and the reported nursing diagnoses, the connection was identified in only 2.3% (*n* = 1).

Concerning nursing prescriptions, the majority (69.7%) were partially present in the nursing records. Only 9.3% of the nursing prescriptions were related to the goals and outcomes described in the nursing record.

The implementation step was rarely recorded, as only 2.3% (*n* = 1) of the nursing records described this item. Finally, in the evaluation step, the review of the plan/nursing evolution was present in 13.9% (*n* = 6) of the records.

**Fig. 2:**
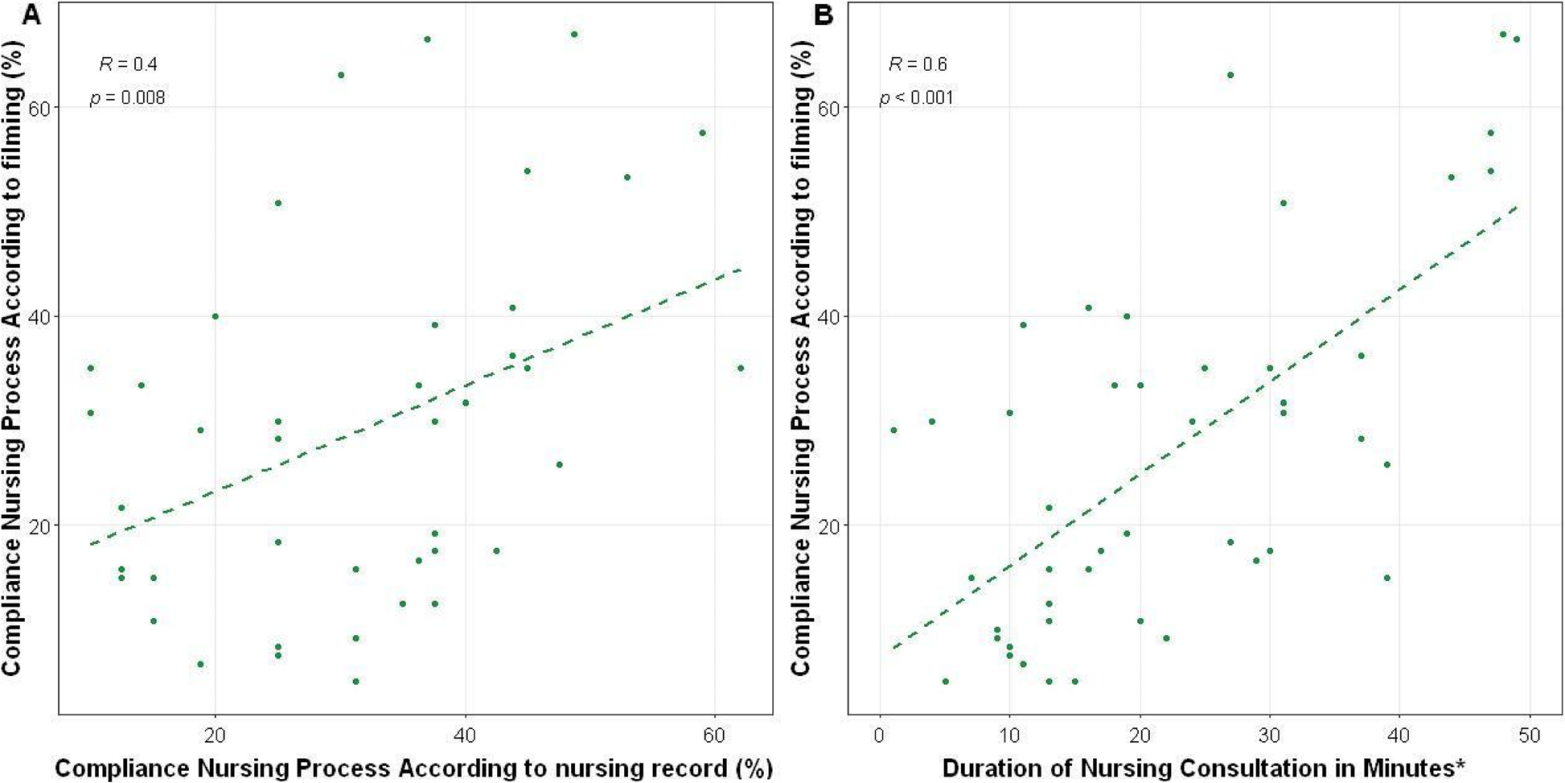
Compliance with nursing process steps according to nursing record and duration (in minutes) of consultation. Brazil, 2023. Note:*Consultations with a duration longer than 50 min were not included

Figure 2 illustrates the compliance with nursing process steps according to the nursing record and duration (in minutes) of the consultation. Graph A indicates the relationship between compliance with the nursing process in the videos (performing the consultation) and the execution of the nursing process in the medical record (documentation of the consultation). Graph B shows an increasing correlation between consultation time and compliance with the steps of the nursing process.

**Fig. 3:**
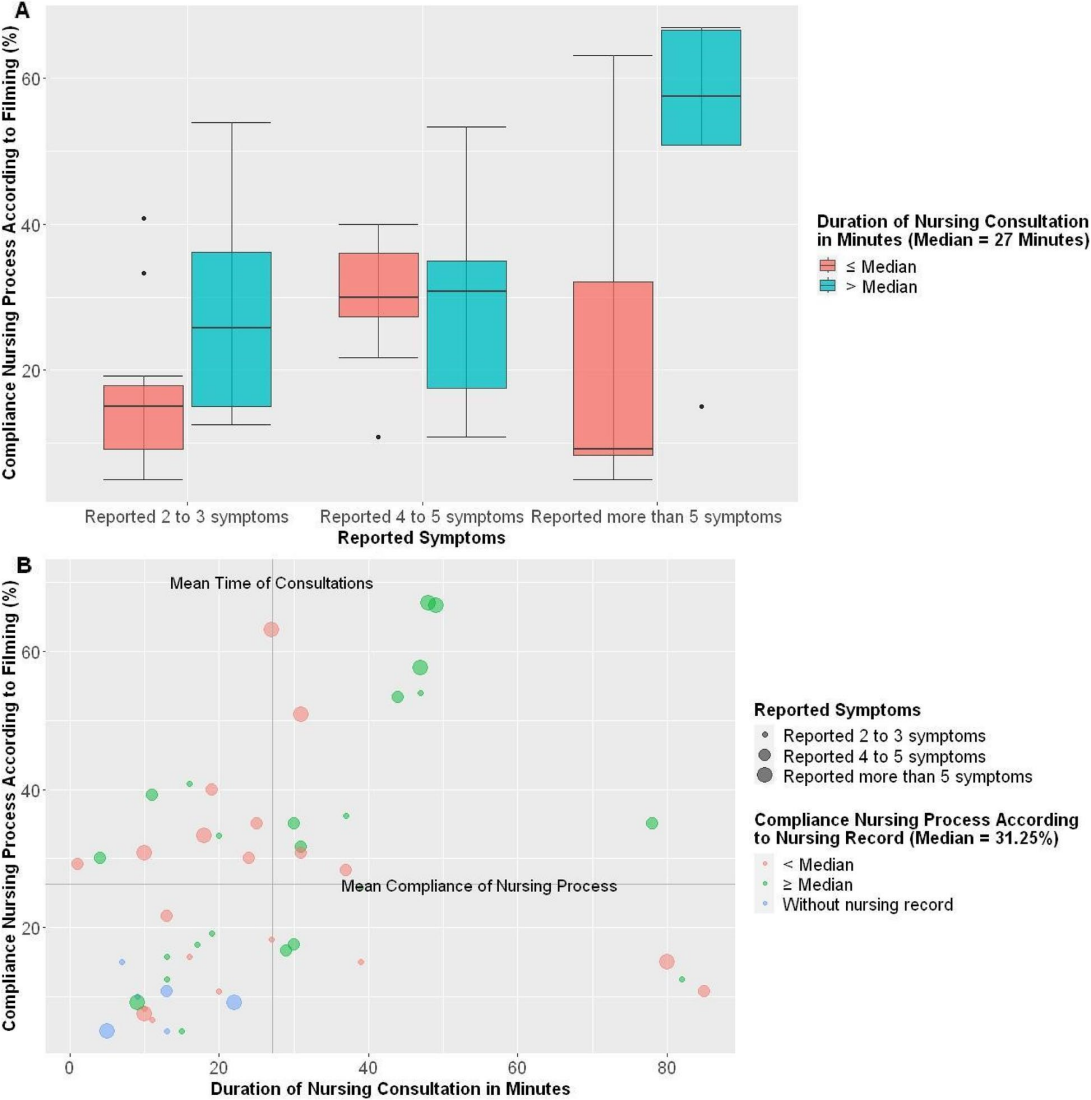
Relationship between nursing process compliance, symptoms, and consultation time. Brazil, 2023

Additionally, the increasing correlation between the consultation time and compliance with the steps of the nursing process remained even after including the number of symptoms, Graph A (Fig. 3). Furthermore, nurses who took nursing process courses in the last year achieved consultations with greater compliance with the nursing process and a longer average time, Graph B (Fig. 3).

Considering the quantitative data analysis, seven consultations were selected that obtained a score greater than or equal to 50% of compliance with nursing process steps (Table 2) and communication aspects to conduct a qualitative investigation into competences related to Advanced Nursing Practices.

**Table 2:** Compliance with nursing process steps according to analysis of medical records and video recordings. Brazil, 2023

Nursing Process Steps Compliance	n(%)
Percentage of completion of nursing process steps based on nursing records (5 dimensions evaluated^a^)	
< 50% compliance	20 (46.51%)
≥ 50% compliance	23 (53.49%)
Percentage of completion of the nursing process steps based on video recordings (6 dimensions evaluated^b^)	
< 50% compliance	42 (85.71%)
≥ 50% compliance	07 (14.29%)

^a^Dimensions evaluated: history, nursing diagnosis, planning, implementation, and evolution

^b^Dimensions evaluated: history, nursing diagnosis, planning, implementation, evolution, and communication

The highest-rated consultations in terms of compliance with nursing process steps were conducted by three nurses from the municipality D. Two of them mentioned having an exclusive room for care and having over ten years of experience in PHC. All reported receiving more than seven minimum wages and one had another job outside the PHC setting.

In addition, each individual holds one or more postgraduate degrees, such as specialization in PHC (*n* = 1), specialization in Family and Community Health, Public Health (*n* = 3), academic master’s degree (*n* = 1), and in another area (*n* = 2). Furthermore, these nurses took at least one training program in the last year in the nursing process; furthermore, one had also taken training in mental health. However, despite presenting the best performance among all those analyzed, most of them responded that they had some difficulty in carrying out the nursing consultation (*n* = 2).

The seven users filmed in consultations presenting greater compliance with the basic elements of the nursing process were female and lived in urban areas. Regarding mental health complaints, all users reported having a diagnosis of anxiety and/or depression. One of the users mentioned another unspecified mental health diagnosis and reported the use of psychotropic medication.

The distribution of symptoms related to mental health was as follows: five participants mentioned difficulty sleeping, nervousness, discouragement, weakness, and dizziness; four reported crying/sadness, ringing in the ears, and multiple pain symptoms; three mentioned shortness of breath, chest pain, and feeling unwell; and only two reported palpitations.

When considering the domain of care management proposed for Advanced Nursing Practices in PHC [30], some competencies were demonstrated during the consultations, although in a partial form, related to care focus (39.68%), assessment and diagnosis (38.78%), and care provision (47.62%) (Table 3).

**Table 3:** Care management competencies for the Advanced Practice Nurse in PHC in mental health consultations. Brazil, 2023

Competencies	NC 1	NC 2	NC 3	NC 4	NC 5	NC 6	NC 7
*Care management: care focus*							
Incorporates knowledge about cultural diversity and health determinants into the evaluation, diagnosis, and therapeutic management of clients and the evaluation of results	A	A	P	P	P	P	P
Incorporates knowledge about development and life stages, pathophysiology, psychopathology, epidemiology, environmental exposures, infectious diseases, behavioral science and demographics, and family processes when performing evaluations, making diagnoses, and providing therapeutic management	A	A	A	A	P	P	P
Incorporates knowledge of the clinical manifestations of normal health events, acute illnesses/injuries, chronic illnesses, comorbidities, and health emergencies, including the effects of multiple etiologies in the evaluation, diagnosis, and therapeutic management of clients and in the evaluation of outcomes	A	A	P	A	P	A	A
*Care management: assessment and diagnosis*							
Uses advanced evaluation skills to differentiate normal, variations from normal, and anomalies	A	A	A	A	P	P	A
Uses technological systems to collect data on variables related to user evaluation	A	A	A	A	P	A	A
Accurately collects and documents relevant client history at each life stage and family life cycle, using other collateral information if necessary	A	P	A	A	P	P	P
Accurately performs and documents appropriate or symptom-focused physical examinations of clients of all ages (including developmental and behavioral screenings, physical examinations, and mental health evaluations)	P	P	P	P	P	P	P
Identifies health and psychosocial risk factors for clients of all ages and families at all stages of the family life cycle	P	A	P	P	P	P	P
Performs differential diagnosis between acute, chronic, and life-threatening conditions	A	A	A	A	A	A	A
Plans screening and diagnostic strategies making appropriate use of technology as a tool, considering the costs, risks, and benefits for customers	A	A	A	A	A	A	A
*Care management: provision of care*							
Provides consistent care in accordance with what is established in clinical guidelines and protocols	A	A	A	A	P	A	A
Provides care in a manner that respects and promotes cultural diversity	A	P	A	P	A	P	A
Communicates effectively, addressing clinical findings, diagnosis, and therapeutic interventions	P	A	A	P	P	P	P
Determines care options and formulates a therapeutic plan in collaboration with clients, considering their expectations and beliefs, available evidence, and the cost–benefit ratio of interventions	P	P	A	A	P	P	P
Incorporates the principles of quality and patient safety into clinical practice	P	A	A	A	A	A	P
Starts a therapeutic plan, conducting pharmacological and non-pharmacological interventions, treatments, or therapies	P	P	A	A	P	P	P
Prescribes medications within its scope of action (national regulations and protocols/programs)	A	A	A	A	A	A	A
Monitors the client’s progress, evaluating and adjusting the therapeutic plan according to their responses	A	P	A	A	A	A	A
Adapts interventions to respond to the needs of people and families in aging, life transitions, and situations of comorbidity and considering psychosocial and financial situations	A	P	P	A	P	P	A
Develops an appropriate palliative and end-of-life care plan	NA	NA	NA	NA	NA	NA	NA

*A* Absent, *P* Present, *NA* not applicable, *NC* nursing consultation

To provide a deeper illustration of the care management domain, the three following scenes represent the different groups of competencies investigated. The Portuguese text was transcribed by the lead author and then translated into English by a professional translator.

For care focus, the following scene indicates that the nurses incorporated knowledge related to psychopathology, family processes, and other psychosocial factors when performing assessments, making diagnoses, and providing therapeutic management.

***Scene:**** 2507-04-U34-D The nurse listened attentively to the user, revisited the presented demands and her evaluation, and made a proposal for agreed-on care strategies. She interacted through gestures and eye contact, maintaining a direct posture toward the user.*

***Discourse:**** E25: “And would you agree to start treating your sadness? I’ll tell you what I learned from our consultation. I see that you have anxiety, that you have this suffering, and this anxiety makes you unable to sleep, makes the pain more intense. And when we don’t sleep, we have pain, we get irritated, because no one can live with so much pain and so much sleep deprivation. I understand you, especially not having anyone to talk to. I understand that all of this becomes a cycle that’s getting harder to support by yourself, isn’t it? (user nods yes). So would you agree to treat this sadness you feel? (user nods yes). OK! This is important. And I’ll say that the treatment can have several phases, several pillars that we talk about, and medication is the last pillar. Not that it’ll start last, but it’s one of the pillars, and we like to talk about other pillars first, for example, we strengthen the support network, improve the quality of life, quality of sleep. You only live with you and your daughter, isn’t it just the two of you?” U34: “Yeah.” E25: “And are you unemployed at the moment?” U34: “Yeah, it’s only been three months, I’m on unemployment.” E25: “Three months. Oh, on unemployment!” U34: “But I’m going to start, people are already finding jobs for me.” E25: “Ah, it’s about to start. Has it been organized?” U34: “At the company I worked for, the supervisor is already calling me.” E25: “And how do you feel about that?” U34: “I’m happy because it’s money and just leaving the house is good.” E25: “That’s right.” U34: “Yes, I worked, I had my problems, but I tried not to show it, because we have to work, and we don’t need to show our stuff.”. E25: “Ahem, yes! There are things at work that don’t have to be opened, right?” U34: “I worked on the street, I worked with supervisors, you know? Going to the building to look for them, I had a lot of fun, enjoyed myself, and talked. I felt fine, but after the company closed, I’ve stayed at home these days.” E25: “Ah, did you leave because it closed?” U34: “It closed”. E25: “Okay! But it’s good that you have a goal in front of you, right, a perspective for the future, which is to go back to work, because that makes you excited. Even because the financial aspect affects our issues a lot, right?” U34: “It affects, but what can you do, right?”.*

Some signs were identified among the consultations related to the competencies for evaluation and diagnosis dimensions. Nonetheless, the nursing records did not present the same level of precision as demonstrated during the consultation. The physical examination focused on the complaint, and the assessment of mental health received greater emphasis in the videos than in the records, as reflected in the following scene:

***Scene:**** 2207-05-U33-D The nurse begins to assess the patient, who is pregnant, mentioning the procedure she would perform (blood pressure measurement). They are sitting side by side; the user shows signs of restlessness (fidgeting hands and legs) and spontaneously asks the nurse a question. From the doubt presented (about the heart), the nurse begins to investigate the symptom through questions that guide her to perform clinical reasoning and search for the cause. The nurse maintains eye contact with the user and interacts through smiles.*

***Discourse:**** E25: “I’m going to check your blood pressure (…)” U33: “Is it normal to feel a lot of pain in your heart?” E25: “Heart pain? How is this pain in your heart like? Explain it to me.” U33: “It’s a twinge.” E25: “Is it always or how frequently?” U33: “I feel it about three times.” E25: “A week, three times a week?” U33: “Yes, and at night.” E25: “Do you feel anything else besides this heartburn, any pain, back pain?” U33: “Yes, I already felt the pain here (points to the heart).” E25: “But when you feel the pain in your back, do you feel the twinge at night?” U33: “The back pain is every day, and the pain is sometimes. I think it’s strange.” E25: “Do you have any torment? Distress? Anxiety?" U33: “I have anxiety.” E25: “And when you have that twinge, do you sense if you are anxious at that moment?” U33: “If I focus too much on that thought, I feel a lot of anxiety and a lot of pain.” E25: “That’s where the pain comes, right? It’s a truly tormented heart. Because look, until now, you haven’t presented any health issues, your follow-ups are very good.” U33: “My blood pressure? I have doubts about my blood pressure.” E25: “Let’s take a look at it. You can lean back, relax. Your pressure is great, it’s 90x60 mmhg”.*

***Nursing record:**** Good general condition, Lucid and oriented in time and space, flushed, hydrated, eupneic in room air.*

*Gestational age: date of last menstrual period 31 6/7; gestational age: Ultrasound 31 2/7; Uterine Height 27 centimeters; Fetal heart rate 136 beats per minute.*

*Position: cephalic; fetal movements present; Edema: no edema; Using ferrous sulfate* + *calcium; Food: eat only 2 meals a day; Did not collect exams from the 2nd quarter; Did not perform a rapid test.*

*Measurements: Weight: 50.9 kg | Blood pressure: 90/50 mmHg.*

*Nursing diagnosis: Ineffective control of the therapeutic regimen, referring to failure to collect tests in a timely manner; attitude toward impaired nutritional status.*

*Nursing diagnosis: impaired comfort.*

Clinical communication skills were investigated alongside the nursing process. However, the therapeutic relationship, which is expected in the exchanges of dialogue, knowledge, and understanding between nurse and user, was achieved in only a few consultations, with the highest-rated ones being particularly notable. The following scene provides some expected elements related to competencies for the provision of care, particularly in formulating a therapeutic plan in collaboration with users, utilizing available evidence, and considering the cost-effectiveness of interventions.

***Scene:**** 2807-04-U45-D The nurse provided guidance on the user’s physical health after carrying out data collection and evaluation. Furthermore, she returned to mental health interaction through direct visual contact with the user and gestures.*

***Discourse:**** E29: “And so this part of anxiety, this feeling of anxiety, we need to talk to the doctor, okay? Relate what you told me to what you are feeling, which is this anguish, tearful from time to time, and let’s see if she prescribes any medication. You know that we have a Multiprofessional Team here, okay? If we’re unable to help you with this part, we’ll look for the Multiprofessional Team to provide this matrix support and give you more complete support, okay?” U45: “Uhm.” E29: “But I believe that by talking to her [doctor], explaining everything we talked about today, her [doctor] prescribing the medication, you will improve.” U45: “I’ll be fine.” E29: “But then we’ll decide together with you to see if this is the best treatment for you, okay?” U45: “Okay.” E29: “But I’m going to leave it separately here so we can discuss this situation”.*

Thus, the findings indicated that, in general, nursing consultation in mental health in PHC are still fragile, as is the presence of the care management competences proposed for the Advanced Nursing Practices in PHC.

## Discussion

Nurses working in PHC conduct nursing consultations with users who present psychosocial needs or complaints related to mental disorders. However, this study found that the nursing process and clinical communication are still fragile. The nursing process steps were underdeveloped in the nursing records, with the focus limited to physical examination during the consultation. Finally, the Advanced Nursing Practices competencies proposed for PHC were poorly identified and, when present, were incipient in mental health consultations.

The profile of users who participated in this study supports the findings in the literature regarding the epidemiological aspects of mental disorders, which predominantly affect women, people of mixed race or black, and the unemployed [29–31], as well as the expressive percentage of anxiety and depression disorders [32].

The complaints and reasons for consultation presented by healthcare users did not initially include mental health issues as the primary reason/complaint. Moreover, when mental health issues were mentioned at any point during the consultation, these were not incorporated into the nurses’ care practice. Considering the burden of mental disorders [32] and the strategic role played by PHC in addressing these conditions, every nursing consultation, regardless of the user’s complaint, should be considered an opportunity for mental health care. Therefore, efforts to improve the nurses’ psychosocial approach are essential to consolidate mental health care in PHC settings.

Although the assessment, management, and longitudinal follow-up of mental health conditions are core elements of this care [25], nurses did not feel comfortable and faced barriers, as evidenced in other studies [33–36], in addition to presenting critical issues in managing human responses. These issues were particularly evident in the fragility of data collection, a key step in the effective development of the nursing process, with care centered on the user’s needs.

The present study found that mental health consultations, as well as those with the highest-rated compliance with nursing process steps, were concentrated in a single study location. This may be explained by incentives in training and the organization of PHC services tailored to meet the demand for mental health care in a specific municipality. This scenario emphasizes the gap in the practice and scientific evidence of this theme within the Brazilian context and the urgent need to address this discussion on a national scale. According to a recent review [34], the studies found in Brazilian literature are mostly qualitative and reflective, identifying challenges and practical implications to psychosocial care.

To improve mental health care in primary care settings, efforts need to be continuous, integrated, and address both micro- and macro-political levels. The mental health working group of the European Forum for Primary Care (EFPC-MH) prioritized 14 themes to strengthen mental health in primary care, with education as a key element in this process. Educational investments must address the entire population to enable prevention, early detection, and address stigma [37]. When considering the workforce, mental health should be broadly integrated into the educational curriculum during undergraduate studies, postgraduate training, and continuing professional development [37].

We suggested that mental health clinical training (undergraduate internship) programs be available in primary care settings in addition to specialist services commonly covered in the nursing curriculum. This would support the recognition of PHC as a component of mental health care, breaking the stigma of mental disorders and helping to develop the necessary competencies to perform nursing consultation and other interventions in this setting.

In the context of professional practice, clinical experience can play an important role in developing nurses’ skills, helping them feel more confident when working with individuals with mental health conditions through experience-based knowledge acquired over the years [38]. However, the evidence suggests that nurses still seem restricted to welcoming and referring the user, based on a biomedical model [34].

Furthermore, stigma is another challenge to overcome. A study carried out in South Africa analyzed the stigma, knowledge, and attitudes of nurses in PHC toward mental health care. Although these nurses provided assistance to users with mental health demands in their activities, most of the participants reported that they had not received training in mental health and had limited knowledge about the subject [39]. Furthermore, that study found that a significant proportion of nurses were inclined to have negative attitudes (stigmatizing) toward people with mental disorders [39], highlighting the importance of disseminating knowledge among primary care professionals to address the mental health gap.

Hence, multimodal interventions are promising. For example, the mental health in PHC project [40] recently implemented in Brazil proposed the combination of two multifaceted strategies: the Health Care Planning methodology and training of healthcare providers for the use of MhGAP-IG [25]. This project achieved encouraging results with the potential for replication in different contexts.

Only one of the studied municipalities (also where the highest-rated consultations were identified) had a structured program for strengthening mental health care in PHC based on the training of healthcare providers to use the MhGAP-IG. The delay in implementing protocols, guidelines, and other institutional initiatives to improve mental health care could limit actions and strategies available to PHC teams. This discussion indispensably encompasses obstacles at broader levels in the political, economic, and social spheres. Thus, despite the progress made in restructuring the mental health care model with the implementation of territorial and community-based care services in Brazil [3], major efforts are still needed to scale up care and reduce the mental health gap.

International experiences provide designs and models for Advanced Nursing Practices to work in the area of mental health, such as the experiences developed in the USA, where, for over 60 years, nurses have been regulated to work as specialists in psychiatric mental health Advanced Practice Nursing [20]. Furthermore, in the United Kingdom, the specialty of nurse consultant roles in mental health services has seen growth since the role was recognized in the 1990s [41]. and other experiences are found in Germany, China, Switzerland, Austria [42], Spain [43], and Canada [20].

The Advanced Practice Nurse is not characterized as a generalist practice but rather as an advanced approach carried out autonomously, with a focus on decision-making skills and clinical competencies [21, 44, 45]. The expected profile of this professional involves critical and abstract reasoning, which includes the initial assessment of users, families, and communities under their care, considering the health problems affecting them, the effective indication of therapeutic alternatives, and continuous monitoring through follow-up and re-evaluation. This practice goes beyond merely adhering to nursing protocols established in national or local health policies [21, 44, 46, 47].

For example, some functions and activities carried out by psychiatric mental health Advanced Nursing Practice or nurse consultant roles in mental health services include psychotherapeutic activities [45, 48] (which may be brief therapeutic interventions), medication prescription, screening, psychoeducation, management of care plans, evaluation and treatment of substance use [49, 50], relating mental health problems with clinical comorbidities [42]_,_ and enhanced communication [43, 45]. Education-related activities are also part of this scope [41, 45, 48].

In clinical practice, Advanced Nursing Practice means that nurses have a greater framework of knowledge and techniques, so that they can legally evaluate, diagnose, prescribe, refer, and carry out care plans. Its differential is greater autonomy in decision-making and problem-solving, enabling the population to access excellent professionals and health services [21, 44]. Furthermore, this role of advanced practice can be enhanced with interprofessional and population-based practice, considering that PHC in Brazil is organized into teams known as family health teams, which coordinate the provision of health care based on the characteristics and needs of the population assigned to the specific location [51].

PHC Advanced Nursing Practice, focused on addressing mental health needs, is not intended to overlap with the existing functions of the generalist nurse in PHC. Considering the positive aspects of the Brazilian context, the implementation of Advanced Practice Nursing and the collaborative role between specialists and Advanced Nursing Practice in PHC is expected to achieve greater resolution of users’ needs and improved access to qualified professionals.

The competencies in care management skills for Advanced Nursing Practice in PHC were observed in only a few nurses who conduct mental health consultations, and these skills remain limited and inconsistent. These nurses presented differences in their consultations, such as the variable time, conducting interconsultation, agreeing on the care plan, taking mental health training, and ensuring continuity of care. In most consultations, they reported the need for a follow-up meeting.

To advance mental health care in order to reduce the existing gap in the health system, investments in the development of essential skills are necessary, such as knowledge about cultural diversity and health determinants, evaluation skills to differentiate normal and variations, accurate collection of users’ relevant history at each stage of life and family cycle, documentation of physical examination (including mental health evaluations), provision of care in accordance with clinical guidelines, and implementation of a therapeutic plan with non-pharmacological interventions.

Investments in developing courses for Advanced Nursing Practice training in PHC in the Brazilian context, whether in a residency and/or professional master’s format [21], will require a high degree of dedication to skills related to using advanced evaluation for differentiating normal, variations from normal, and abnormal; using technological systems to collect data on variables related to user evaluation; carrying out differential diagnosis between acute, chronic, and life-threatening conditions; screening and diagnostic planning and strategies; making appropriate use of technology as a tool that considers costs, risks, and benefits for users; offering consistent care following established clinical guides and protocols; realizing quality and safety principles; prescribing medications; monitoring the user’s progress, and evaluating and adjusting the therapeutic plan according to their responses.

Furthermore, to achieve competencies within the scope of mental health in PHC, a nurse must understand that commitment and involvement in the encounter with the user, the multiple dimensions of the life history, and social, cultural and economic contexts help expand identification of needs that are often not seen by the user themselves as factors of illness and/or are not valued by the professionals themselves. Together, the development of skills for managing emotional aspects, the inclusion of the user in the decision on care strategies, and the sharing of actions with other team members also stand out.

Therefore, considering the different functions performed by PHC nurses, which range from responsibility for managerial activities to care activities, and the range in which they perform different skills, their work must have greater consolidation with more defined assignments that facilitate the performance of advanced roles in their practice [10], so that the population can have greater access to mental health care.

In the face of the broad health care needs of the population, such as those related to mental health, the development and integration of Advanced Nursing Practices into the healthcare system can improve the population’s access to qualified health professionals [5, 52]. Nonetheless, in the Brazilian context, some challenges for implementing are related to a biomedical health model, resistance from some professional categories, regulatory legislation, and lack of government incentives [43, 53]. Internationally, some factors for the implementation of the Advanced Nursing Practice corroborates the Brazilian context, including education, regulation, organizational environment, interprofessional collaboration, and lack of clarity about the role [54]. On the other hand, Brazil is one of the Latin American countries with the favorable conditions for implementation, due to its growing advancements in nursing education, highlighted by the number of postgraduate programs, as well as regions that have a lack of labor and difficult access, which would promote greater health coverage [21, 43, 44].

### Limitations

The limitations of this study are related to the potential change in expected behavior during the methodological process of filming the consultations, which may cause shyness and embarrassment for both nurses and users. Additionally, the sample size and selection may underestimate the measures due to selection and classification bias. However, this investigation provides significant results to support the discussion on the topic with institutions responsible for nurse education, professional organizations, local management, and especially nurses working in PHC.

Although the discussion around Advanced Practice Nursing has been going on internationally for over 50 years, in Brazil, it is still a relatively recent topic that requires robust research to support and identify the best pathways for Advanced Practice Nursing in the Brazilian Unified Public Health System (SUS), which is centered on PHC. Moreover, it is crucial to advance implementation research to explore how countries like Brazil can leverage the role of nurses as essential professionals in ensuring an effective response to the population’s mental health needs.

## Conclusion

The findings reveal that PHC nurses lack training through permanent and continuing education to develop skills to intervene in psychosocial care, reinventing their practice and expanding their autonomy. Furthermore, nurses who carry out mental health nursing consultations in PHC present, in a scarce and partial way, the competencies proposed for the Advanced Nursing Practice regarding the domain of care management. Therefore, it is recommended that training institutions expand their curricula and invest in mental health care training for PHC nurses to enable these professionals to make clinical decisions, increasing the population’s access to qualified mental health care, valuing the profession, and building regulations for the training of Advanced Practice Nurses in Brazil.

## Data Availability

The datasets used and/or analysed during the current study are available from the corresponding author on reasonable request.
